# A dynamic systems view of clinical genomics: a rich picture of the landscape in Australia using a complexity science lens

**DOI:** 10.1186/s12920-021-00910-5

**Published:** 2021-02-27

**Authors:** Janet C. Long, Hossai Gul, Elise McPherson, Stephanie Best, Hanna Augustsson, Kate Churruca, Louise A. Ellis, Jeffrey Braithwaite

**Affiliations:** 1 Australian Institute of Health Innovation, Macquarie University, Sydney, Australia; 2 Australian Genomics, Murdoch Children’s Research Institute, Melbourne, Australia; 3 Murdoch Children’s Research Institute, Melbourne, Australia; 4 Medical Management Centre, Department of Learning, Informatics, Management and Ethics, Karolinska Institutet, Solna, Sweden

**Keywords:** Complexity, Health services research, Implementation, Sustainability

## Abstract

**Background:**

Clinical genomics represents a paradigm shifting change to health service delivery and practice across many conditions and life-stages. Introducing this complex technology into an already complex health system is a significant challenge that cannot be managed in a reductionist way. To build robust and sustainable, high quality delivery systems we need to step back and view the interconnected landscape of policymakers, funders, managers, multidisciplinary teams of clinicians, patients and their families, and health care, research, education, and philanthropic institutions as a dynamic whole. This study holistically mapped the landscape of clinical genomics within Australia by developing a complex graphic: a rich picture. Using complex systems theory, we then identified key features, challenges and leverage points of implementing clinical genomics.

**Methods:**

We used a multi-stage, exploratory, qualitative approach. We extracted data from grey literature, empirical literature, and data collected by the Australian Genomic Health Alliance. Nine key informants working in clinical genomics critiqued early drafts of the picture, and validated the final version.

**Results:**

The final graphic depicts 24 stakeholder groups relevant to implementation of genomics into Australia. Clinical genomics lies at the intersection of four nested systems, with interplay between government, professional bodies and patient advocacy groups. Barriers and uncertainties are also shown. Analysis using complexity theory showed far-reaching interdependencies around funding, and identified unintended consequences.

**Conclusion:**

The rich picture of the clinical genomic landscape in Australia is the first to show key stakeholders, agencies and processes and their interdependencies. Participants who critiqued our results were instantly intrigued and engaged by the graphics, searching for their place in the whole and often commenting on insights they gained from seeing the influences and impacts of other stakeholder groups on their own work. Funding patterns showed unintended consequences of increased burdens for clinicians and inequity of access for patients. Showing the system as a dynamic whole is the only way to understand key drivers and barriers to largescale interventions. *Trial Registration:* Not applicable

**Supplementary Information:**

The online version contains supplementary material available at 10.1186/s12920-021-00910-5.

## Background

Informed by systems theory, the case has been made that the health system is a complex adaptive system (CAS) [1, 2]. This is in contrast to a view that, although very complicated, the individual parts of the system work in a linear, mechanistic way with high predictability of outcomes [2, 3]. Alternatively, CASs are characterised by multiple, semi-autonomous agents that interact, have interdependencies and tend to self-organise; these systems therefore display emergent behaviour, and have non-linear, unpredictable outcomes. Health systems fit this model well with their large numbers of interacting health professionals, patients and family members, nested departments, specialties and services, self-organising teams, and social processes. In such a system, focussing on the individual parts in isolation will not lead to an understanding of the system as a whole and is inadequate to address challenges that arise [4]. For example, the slow uptake of evidence into practice has been linked to a reductionist, linear “pipe-line” approach that fails to take into account local contextual constraints or interdependencies [3, 5]. Silos of professional groups, departments, or disease type have been associated with a number of intractable problems in healthcare, such as lack of integration of services [6], poor communication [7], unhelpful gatekeeping of information [8], and resistance to change [9, 10]. Yet silos are known to be a naturally emergent feature of complex systems [11, 12] and have strengths as well as weaknesses [13]. Understanding these system features by using a complexity theory lens will increase our ability to intervene and leverage improvements [14, 15].

It is becoming increasingly apparent that we need to deepen our understanding of health systems around the globe as they introduce disruptive technologies such as genomic testing into already complex systems [16]. Particularly pressing is the gap in our knowledge of complexity within largescale translation initiatives such as the implementation of clinical genomics. A major player in this endeavour within Australia is the Australian Genomic Health Alliance (hereafter Australian Genomics). This is a transdisciplinary national alliance consisting of over 400 clinicians, pathologists, clinical and basic research scientists, non-medical multidisciplinary researchers, and community representatives, performing translation activities across 30 varied sites, conditions, and contexts [17]. ‘Flagship’ clinical projects throughout Australia have collected data on clinical utility, patient satisfaction and feasibility across a wide range of rare diseases, genetic syndromes and cancers.

Implementation of clinical genomics requires major changes across the health delivery system. Use of this new technology requires new laboratory equipment and processes, enhanced data storage and sharing for the gigabytes of data each patient’s test generates, and changed interdisciplinary team configurations and practices. This represents a significant learning curve for those involved and necessitates cultural shifts around ways of working; e.g., moving from single patient to family focussed care.

This study complements a larger program aimed at developing our understanding of various aspects of the complexity encompassing the translation of genomics into routine care. The larger study contains a scoping and critical review of genomic implementation literature analysed via a complexity lens [18], and a longitudinal Social Network Analysis (SNA) of Australian Genomics to describe patterns of socio-professional interaction [19].

The aim of this study was to develop a ‘rich picture’ through collection and analysis of a range of data to map the landscape of clinical genomics within Australia, allowing a more holistic evaluation of the translational activities of Australian Genomics. The rich picture was then analysed using complex systems theory to identify key features of the system, reveal challenges to progress, and suggest leverage points to supplement genomic implementation efforts.

### Rich pictures

Rich pictures are a tool used in Soft Systems Methodology [20–22] to define and describe a complex human situation through drawings or diagrams. The rationale for using pictures to describe complex human situations is that such situations entail multiple interacting relationships which are not easily captured in tables or in written or spoken language. To develop a rich picture, information about the situation is gathered, for instance, by interviewing individuals with an understanding about the situation, attending meetings and by reviewing documents. The gathered information is then used to develop a graphic depiction, which elucidates the links between the different structures, roles and viewpoints of a situation, as well as the processes going on and current and potential future concerns [22, 23]. Thus, the use of rich pictures helps to visualise the interrelationships and influences both within and between parts and levels of a system. This in turn, helps to view systems as wholes rather than looking at parts of systems in isolation. As such, rich pictures are an ideal way to capture elements of a complex adaptive system [24]. While acknowledging that no static graphic can capture all the elements of the system, or completely represent the emergent properties of these elements, we chose a rich picture as the basis for our study, and developed a narrative to accompany it.

The rich picture method acknowledges the role of human behaviour driven by things other than on-the-surface logic (e.g., peer pressure, anxiety, ignorance). It does not assume, for example that everyone follows the rules unquestioningly, or behaves rationally. This gives freedom in the data collection phase to include uncertainties and contextual factors that might not otherwise be apparent. Such complex human situations are a characteristic of a CAS in the context of health care. Specifically, social interactions such as group decision-making, learning-by-doing, and collaboration are key processes in clinical genomics (e.g., for multidisciplinary curation teams), which can in turn introduce uncertainty, unintended outcomes, feedback loops and dependencies.

## Methods

**Fig. 1:**
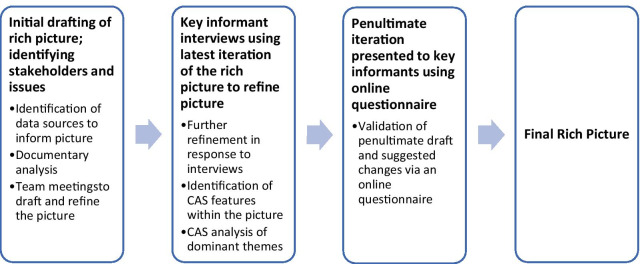
Overview of the procedure used to develop and ratify a rich picture of genomic translation in Australia. (CAS = complex adaptative system)

A multi-stage, exploratory, qualitative approach was used to develop the rich pictures. Starting with a list of stakeholders and issues known to our team from previous work in clinical genomics [16, 19, 25, 26], we constructed the first iteration of the rich picture to illustrate the interplay of factors around the introduction of clinical genomics into healthcare in Australia. Informed by the document search and analysis, researchers (JL, HG and EM) used a white board to sketch out the initial picture for discussion within the team. Then as it was refined, electronic versions of the rich picture were used to experiment with layout and facilitate accurate depictions of interactions. *Creately* software [27] was used to generate the picture. As interview data were collected, they were incorporated into the rich picture. Figure 1 provides an overview of the procedure. Validation of the penultimate version was conducted via an online survey. Refinements from this round of data collection produced the final version of the picture.

### Data

Data from a variety of sources were collected to develop the rich picture exploring how clinical genomics was being used and developed within Australia including: Australian Genomics documents (website, Flagship project protocols, reports) and organisational reports (e.g., number of genetic counsellors, number of postgraduate students), websites (State and Federal Government Health Departments, professional and regulatory bodies, patient advocacy websites). The research team evaluated the various data sources looking for stakeholders to include in the picture, and interactions between the various components. Findings were structured around broad issues such as funding, workforce capacity, health and laboratory services, infrastructure, equity, influence of professional and regulatory bodies, and policy at the national level. Issues were explored considering how clinical genomics was being used and developed within Australia. More detail was added to each broad issue iteratively, and the interactions between issues within the system were mapped. A set of literature compiled as part of a systematic literature review [in preparation] on the implementation of clinical genomics was used to identify issues from the broader, global genomic field that were relevant to Australia, and also informed the picture.

Medical genomics maps across many areas of the health service as it is practised over a broad range of specialist fields and life stages. Outside of, and interacting with the service, our graphic mapped the broader health system. Research and educational institutions, professional bodies, government departments, insurance agencies, biotechnology industry, and consumer groups were all found to have a role and active interest in genomics, broadening our graphic’s boundary. CASs by definition have “fuzzy boundaries” so we used a pragmatic approach to this by identifying all stakeholders at the micro, meso or macro level that had a role, or potential role in medical genomics and including them. We also specifically asked our participants whether there were parts of the system that we had left out to ensure our graphic was as comprehensive as possible.

### Semi-structured interviews

Ten people integrally involved in clinical genomics through Australian Genomics or partner organisations were invited to be our key informants and to contribute to the project by commenting on the rich picture in two rounds of consultation; the first consisting of a semi-structured interview commenting on an early iteration of the picture; the second round commenting via an online questionnaire on the penultimate version of the picture. Key informants held different roles in clinical genomics (e.g., health services, clinicians, laboratory, education, national level management) to ensure the main stakeholder groups were represented.

Key informants were identified and approached by an embedded researcher (SB) within Australian Genomics; those who indicated interest in participating were followed up by the external researchers. All participants were given information about the project and were required to give written consent for the first-round interview to be audio-recorded. Audio recording allowed researchers to capture, in the participants’ own words, how people working within clinical genomics frame and explain key processes. Interviews were conducted face-to-face at a mutually convenient time and venue by health services researchers JL, EM and HG with qualitative research expertise.

The interview had two parts; consideration of the picture, and commenting on three dominant themes that emerged during the planning phase of the project. During the first part of the interview, participants were shown the early version of the rich picture and given time to study it. They were informed that it was a graphic of features, influences and stakeholders involved in Australian Genomics’ endeavour to introduce genomic medicine into routine care in Australia. They were given a pencil and encouraged to draw on the graphic to add, subtract or move items and to “think out loud” while doing so [28]. Specific questions asked were: *Do you think this is an accurate representation of clinical genomics at the national level? Have we missed any components or stakeholders? Have we missed any interactions or influences?*

During the second part of the interview, participants were asked about the three themes: (i) funding for genomic testing; (ii) how genomics necessitates new ways of working; and iii) how genomic medicine can give rise to unpredicted or unintended consequences. These themes were identified from findings of the scoping and critical review of implementation, and previous interviews as issues that are strongly linked to the practice of genomics, have a high impact on implementation outcomes, and generate much discussion. Questions were open-ended, stating the theme (funding, new ways of working, and unintended or unpredictable consequences) then asking for the participants’ thoughts. Interviewees were not directly prompted to identify or discuss features of complexity such as feedback loops or interdependencies. Interview schedule is supplied as Additional file 1.

### Analysis

Data from the first part of the recorded interviews together with the pictures the participants drew on and amended were used to further develop the rich picture; moving components around, adding interactions, feedback loops and barriers, or additional stakeholders. This was done by one researchers (HG) and then discussed and ratified by the larger team.

We then interrogated the components and interactions within the resulting rich picture individually and collectively for evidence of complexity using a framework of features associated with CAS. The framework used was developed and adapted from our previous work in complex systems [3, 29–31]. Two researchers (HG and JL) undertook this work before discussion and refinement with the larger team. In the next step, the three themes of interest (mentioned above) from the second part of the recorded interviews were transcribed and analysed. Data was coded using the same framework of CAS features as used above. Coding was undertaken by three researchers (HG, EM and JL) and then discussed, refined and validated with the larger team (SB, HA, KC, LE, JB). Wherever possible, CAS features from the interviews were also added to the rich picture. This provided more detail and illustrative stories around components in the rich picture. The evidence gained from the study was compiled and ways we could leverage naturally emergent network phenomena and strategically drive useful outcomes were considered.

### Ethics and governance

This work was funded by Australian Genomic Health Alliance and the Murdoch Children’s Research Institute. It received approval from Macquarie University Human Research Ethics Committee (Ref: 5201701186) and was endorsed as an Australian Genomics member activity by the executive.

## Results

**Fig. 2:**
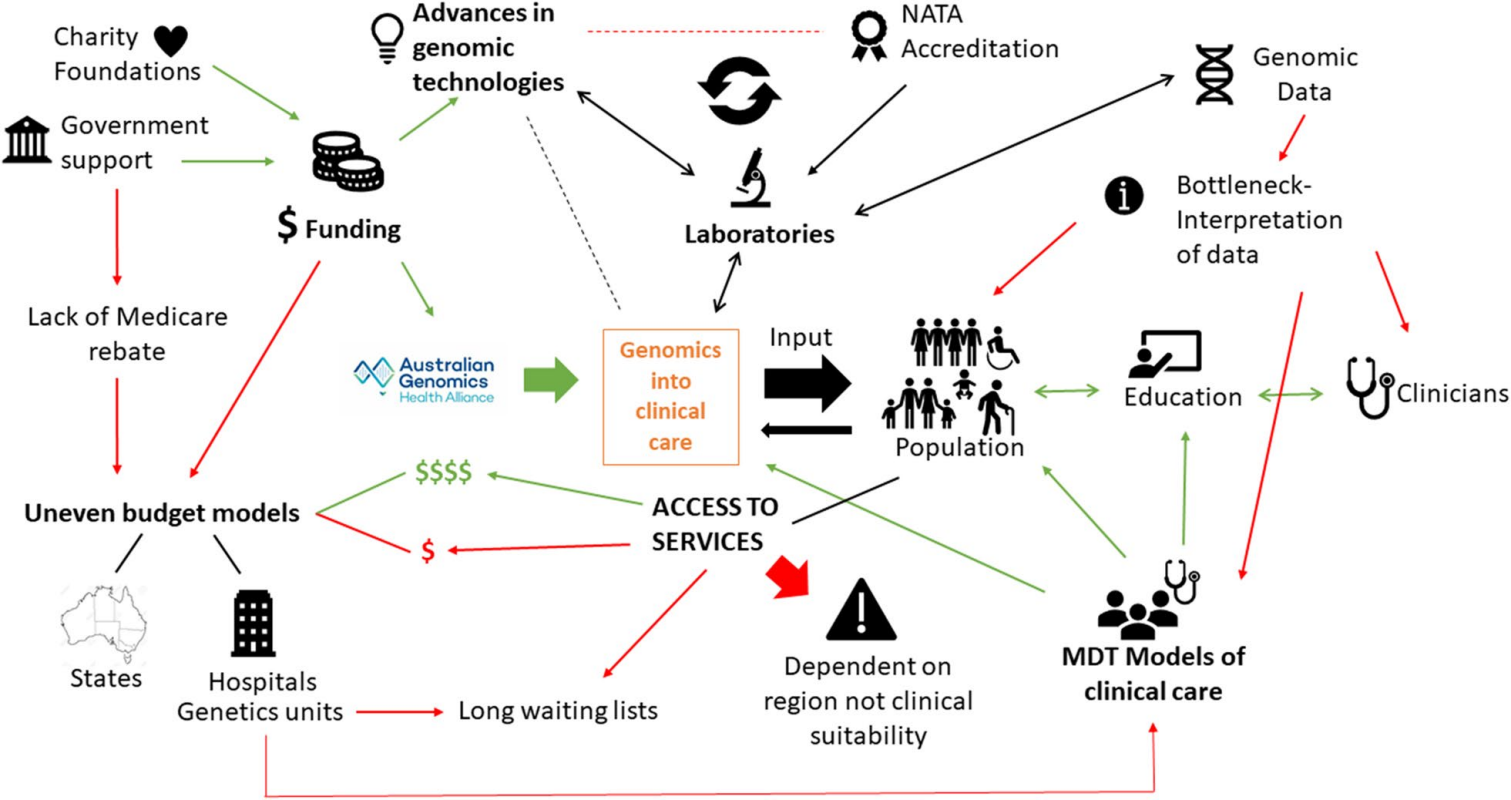
Rich picture iteration #3 used for first round of key informant interviews. Copyright permission to use Australian Genomics logo was obtained

Sixteen iterations of the rich picture were generated. Iterations #1–3 were developed using Australian Genomics documents, grey literature, and our systematic review. The graphic used for the key informant interviews (Iteration #3) is show in Fig. 2. Nine of the ten invited key informants agreed to participate in interviews, resulting in 333 min of data (average of 25–35 min each). Table 1 shows details of the participants.

**Table 1:** Characteristics of participants involved in interviews and feedback on rich picture

Key Informant ID	Organisation type	Role in genomics	Area of interest to us (e.g., patient attitudes, funding)
KI1	Research Institute	Health services researcher	Flagship processes, service level processes
KI2	Laboratory	Medical science liaison	Laboratory processes, links with industry
KI3	Clinic	Genetic counsellor	Genetic counselling, patient attitudes, access to services, models of care
KI4	Clinic	Genetic counsellor	Genetic counselling, patient attitudes, access to services, models of care
KI5	Research Institute	Administrator	Infrastructure and supportive work
KI6	Research Institute; hospital	Research assistant and research genetic counsellor	Recruitment of patients into genomic research, patient perspectives, clinical processes
KI7	Australian Genomics	Research Manager	Overview of programs and working parties within Australian Genomics
KI8	Research Institute; hospital	Clinical lead and researcher	Involved in a number of programs and flagships; international experience of genomics
KI9	Research Institute; hospital	Clinical lead and researcher	Involved in a number of programs and flagships; international experience of genomics

### Rich picture: overview

**Fig. 3:**
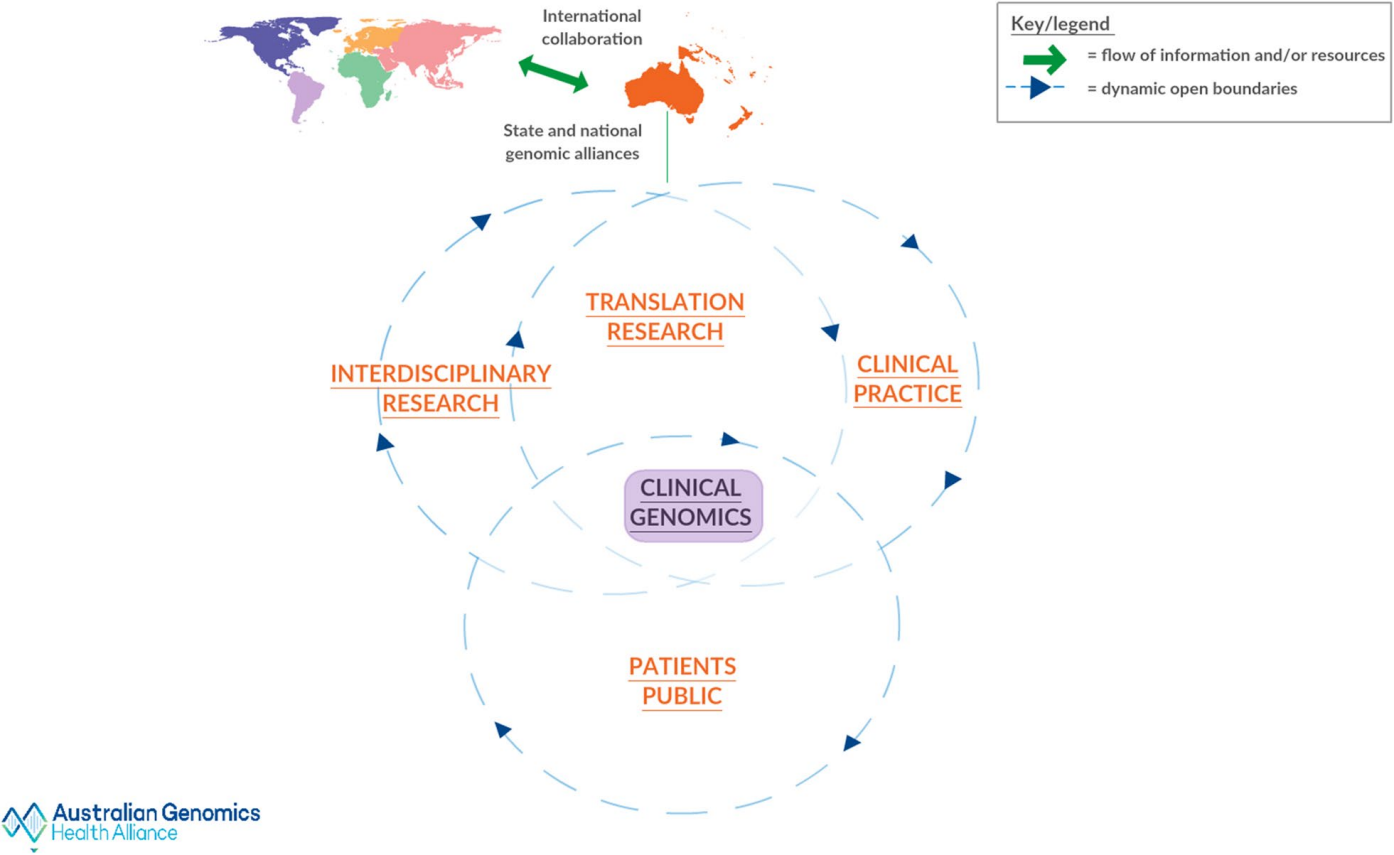
Nested systems at the centre of the final iteration of the rich picture. Copyright permission to use Australian Genomics logo was obtained

The final rich picture is structured around four nested systems (Fig. 3): Interdisciplinary Research, Translational Research, Clinical Practice, and Patients/Public. In the centre of the picture, at the intersection of these four, lies Clinical Genomics.

**Fig. 4:**
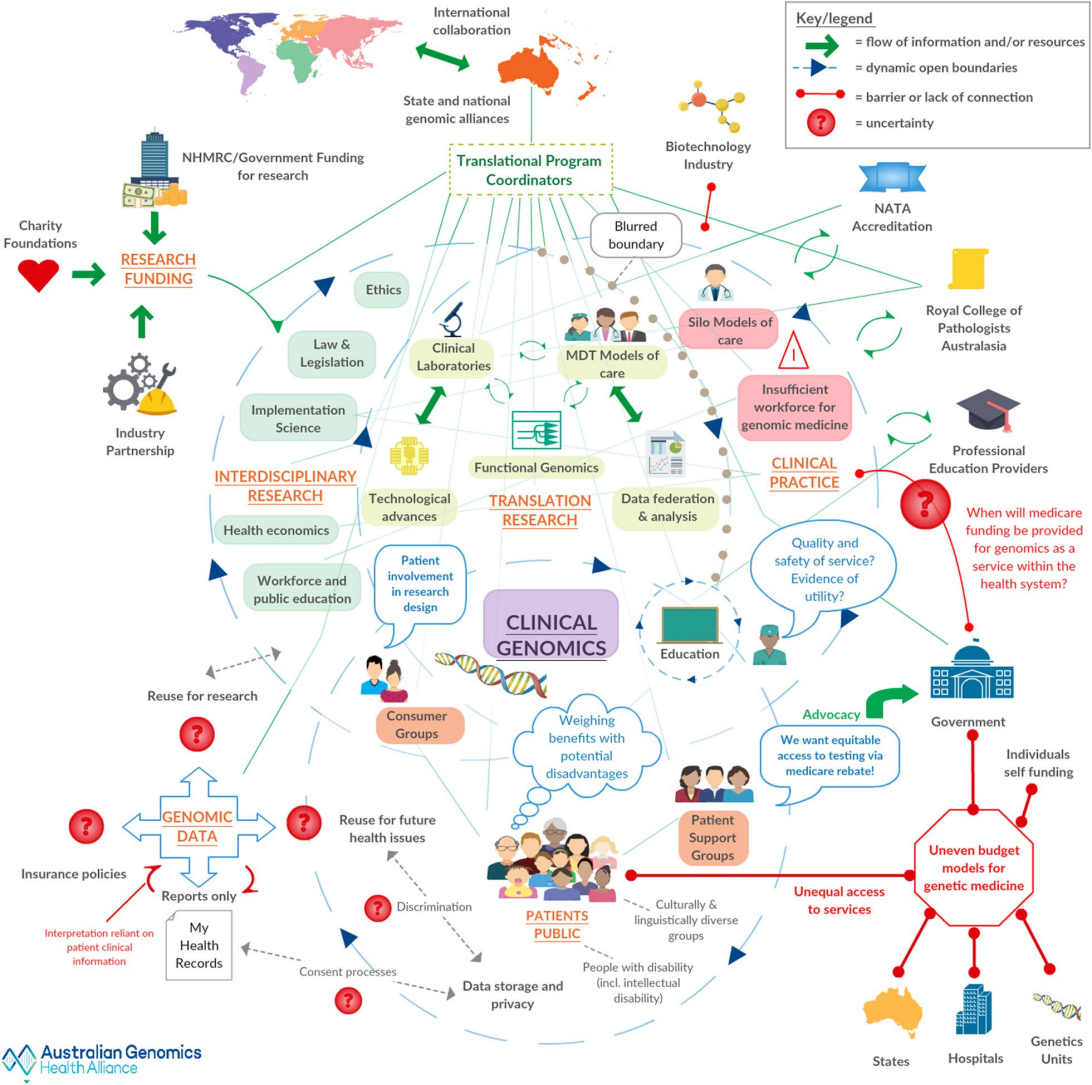
Final iteration #16 of the rich picture of the clinical genomics landscape in Australia. Copyright permission to use Australian Genomics logo was obtained

These systems have open dynamic boundaries acknowledging the overlap of stakeholders, aims and activities as depicted in the final rich picture (Iteration #16), shown in full in Fig. 4. From the key informant interviews and organisational document analysis, a total of 24 types of stakeholders were identified as relevant to the issue of implementing genomics into the Australian healthcare system. These stakeholders spanned multiple levels (e.g., consumer groups, clinicians, researchers, professional bodies, multiple levels of government) and were accompanied by a range of technological artefacts (e.g., My Health Record, data federation and analysis systems).

Translational Research is shown at the intersection of Interdisciplinary Research and Clinical Practice. Within Translation Research there is a dynamic interplay between Clinical Laboratories, Multidisciplinary Models of Care and understandings from Functional Genomics research, all supported by Technological Advances, Data Federation and Analysis. Depicted within Clinical Practice but extending in influence into Translational and Interdisciplinary Research, sit the important issues of Siloed Models of Care and Insufficient Workforce for Genomic Medicine. Within the Patients/Public domain, lie Patient Support and Consumer Groups with key issues of equity of access, privacy, and realisation of benefits of genomic medicine while minimising the disadvantages. The Translational Program Coordinators of Australian Genomics are depicted as sitting on the top of the Clinical Genomics cluster linking all the elements together and liaising with external stakeholders.

Around the edge of the Clinical Genomics cluster are external stakeholders and influences. For example, Government, Charitable Foundations and Industry Partnership (top left) provide funding to drive research. Other external influencers include key professional bodies and education providers, Government and their various budget models and the presence of the growing bank of Genomic Data.

Barriers to the integration of genomic medicine into healthcare across Australia that affect Australian Genomics are shown as thick red lines with each end terminating in a dot. There are a cluster of barriers in the lower right hand section of the picture around uneven budget models involving State and Federal governments, hospitals, Genetic Units and self-funding individuals. These are contributing to unequal access to services. Another barrier is a lack of engagement with the Biotechnology industry (top right).

### Features of a complex adaptive system: rich picture

Some features of a CAS were depicted in the rich picture while others were only apparent through personal experiences of the system, which were described by interview participants. CAS features represented in the rich picture include the porous boundaries with unclear borders between the four nested systems, the many agents at multiple levels including both individuals and organisations, and the web of interactions and interdependencies. There were also numerous uncertainties, which are depicted as red question marks and are mainly clustered in the lower left of the picture. Uncertainties involved Genomic data: access to the data in the future for personal health or research purposes, how consent processes protect privacy and safe data storage, and how genomic testing affects insurance status. At present, there is no availability of Medicare funding (universal insurance for Australian citizens) to access genomic testing, so this is portrayed as both a barrier and an uncertainty for Clinical Practice. Arrows throughout show the flow of information or resources that are facilitating processes. For example, Patients’ Advocacy for a Medicare Item number for genomic testing to the Australian Federal Government.

### Features of a complex adaptive system: key informants’ experience

Features of a CAS were identified in all the key informant interviews. Table 2 summarises the findings, presenting themes found in the interviews and providing exemplar quotes. Most common features discussed were uncertainties, interdependencies and unintended consequences. Uncertainties pertained to ownership of genomic data, availability of future funding for testing, and questions around future demand for testing and the capacity of the workforce, laboratories, and biotechnology industry more broadly to meet that demand.

**Table 2:** Features of complex systems identified in key informant interviews

Feature of complex systems^1−5^	Definition	Issues identified	Exemplar quotes [KI = Key Informant]
Uncertainty	Arises from the inability to predict outcomes in a CAS, or from clinical situations (e.g., around a patient’s prognosis) due to a range of factors, including the number of inputs and lack of knowledge	Uncertainty around who owns the data and how the data will be reused in the future	So many questions. Can patients access their data? Who can have access or store it? What are you going to do with the data? Are you going to sell it? Can insurance companies use the data to discriminate against the person? [KI2]
		Dealing with unexpected findings that do not fit the work plan	There is one size fits all [with no room for variation]. So, for example, what happens when a trio comes in [testing of child and both parents] and the dad is not the dad? [KI2]
		Will the different stakeholders adapt and adopt genomic new technologies and processes?	There’s uncertainty around how we get the different states [in Australia] to adapt and adopt these new technologies and processes and industry—this will be critical. [KI7]
		Uncertainty about demand for genome sequencing	There’s a huge amount of uncertainty; we are building it as we go and trying to get it right.[KI2]
		How will we fund clinical genomics once the research funding is withdrawn?	We do wonder where funding might come from for patients [in the future] [KI4]
Non-linear processes	Occur in complex systems as the dynamic nature of agent interactions, interconnections and emergence means processes cannot be linked causally to outcomes in a linear way	Patients were sometimes unwilling to have genomic tests as they thought it may affect their insurance	Uptake [of genomic testing] on a large scale is affected by non-health and biomedical issues such as insurance. It’s been affecting recruitment and research participation [KI7]
Unintended consequences	Interactions can occur on multiple timescales. As interdependencies increase, so does the likelihood that a given action will generate unintended consequences	Additional meetings and paperwork were necessary to allocate limited funding for tests	The amount of paperwork is mind boggling. I’m spending so many hours on it instead of looking after patients or interpreting data [KI9]
			One of the hardest thing about my job is trying to figure out how to help people access testing. It’s time-consuming. You need to consider what they’re eligible for, how do I make it happen, what paperwork needs to be filled out. It’s a significant part of my job now. [KI4]
			The [genomic] data’s so complex that the lab people can’t analyse it. It started with the clinicians doing all the analysis for about 18 months while the lab were hardly involved in it at all. That was when it was a research project through [State-based genomic project]; it wasn’t an accredited clinical test. When it became clinically accredited it sat much more firmly with the lab but they still needed input for several hours a week and that was not accounted for in either budget [KI9]
		Amount of clinician time spent with laboratory services in data analysis and interpretation was unexpected and contributed to time pressures to return results and budget issues	The model for how the laboratory should work is changing. They’re negotiating that with them and there isn’t a good established model for how that should work in the future. 30% of {Clinician X’s] time is spent helping them analyse cases and that’s not accounted for in the clinical or laboratory budget. It’s been like that for the past 3 or 4 years and remains unresolved. It’s also unresolved in the UK. There’s an increasing need for clinical input in the laboratory as part of laboratory services. The job description/KPI for this hasn’t been created. [KI9]
		Investment in time spent in teaching for the senior clinicians and scientists was unexpected	Upskilling people to be on par contributors to [multidisciplinary teams] has been a challenge. Same thing with [genetic counsellors] and lab people. You need a few years of training to function at the required level. So, lots of supervision in my role has become a new way of working that exists because of genomics. [KI9]
		Clinicians can feel lack of funding undermines their patient care	An issue as a Genetic Counsellor working as an advocate for the patients, if a result comes back … [and it is] suggested as clinically useful and beneficial for other family members to have testing, there’s often no avenue for funding for follow-on testing. Australian Genomics will fund follow-on testing for family members if it aids in firming up classification of a variant as benign to pathogenic … But if it won’t clarify the classification of a variant then they won’t fund that. [KI3]
		Some diagnostic tests/roles may be made redundant as a result of genomic testing	Some of my colleagues are worried about their area of expertise not being valued anymore, for example those who look at X-rays or skilled morphologists. Genomics will replace some tests or skills … That’s now challenged by genomics. Is there a need for that particular skill set at that level? [KI9]
Interdependencies	Interdependence involves the emergence of some overall combined system to the organization, where individuals retain their autonomy with respect to each other, but they are interdependent with respect to the overall combined organization	Nature of the workforce linked to funding which is linked to capacity and experience	It’s a young [Genetic Counsellor] workforce because [with the amount of funding available] they could only afford year 1 counsellors—Junior counsellors straight out of the Masters program… [This has had flow on effects] … [the junior counsellors] get thrown into a situation with very sick children … it’s very hard for them. [KI9]
		Tests are contingent on inclusion and exclusion criteria	If parents are eligible for testing but one parent is deceased, then the testing will not be funded as it is “not fully informative.” [KI3]
		Tests contingent on postcode and budget models rather than on need	There are inequities… [Patients in some regions] may not have access to funding for whole exome/whole genome testing and will need to fund themselves and this is challenging because tests can cost thousands. That’s a huge barrier. It’s not due to lack of clinical or sub-speciality interest. There’s a huge interest and desire to get tests for patients, but there’s not necessarily the funding. [KI4]
		Consumer groups are advocating advances in genomic technologies as the benefits are realised by their members	There are a lot of support groups that are doing advocacy work and lobbying the government … to bring awareness for how much this technology is needed. Patients go online and see that testing is available overseas and start asking why it’s not available here. [KI4]
		Clinicians rather than industry should be driving new technology	Clinicians should be connected to this as they are drivers for getting the testing up and running [in the Flagships] and having the conversations with labs, “what can you do to get us this type of testing?” rather than the other way around. [KI4]
		Various outcomes are reliant on partnerships	When you get to the end of a clinical flagship project funded and you just sort of assume that you're going to throw this new technology or test over the wall, and it's going to be picked up here, but it's not. And what's more, there has been communication, in some contexts like [clinical flagship], where they [industry] overtly say “you involve us at the start, or we will make sure it is not adopted.” [KI7]
Self-organisation/ emergence	Involves creating order from within rather than imposed from outside the group; based on the importance of relationships and influences between agents	New ways of working have emerged between clinicians and laboratory services; working collaboratively rather than separately	A lot of time is now devoted to working with the labs and contributing to data analysis. In the past, we might have been consulted once in a blue moon by the lab about a tricky case – how to report or word something. Now it’s many hours a week sitting with them and looking at data. [KI9]
	Emergent phenomena or behaviours arise from the dynamic system, and are not predictable based on the expected outcomes	New roles are emerging	Bioinformaticians have also entered the lab workforce. The role of the bioinformatician has emerged without a formal accreditation pathway. They come from a research background and they’re university trained. Many of them are developing and operating systems [to assist in genomic result curation]. [KI9]
		Bottlenecks in the genomic testing process have changed	Despite tools to help with interpretation, a lot of it is still manual; people review the literature and tools. Some more complex variants can take up to 20 h of curation…. The time required to fill a high-throughput machine with [genomic] samples used to be a big bottleneck when fewer people were ordering testing. But the space is changing so quickly that that issue has been replaced with new issues, i.e., time for curation…. [join] the queue waiting for the data to be interpreted. [KI4]
Feedback loops	A phenomenon in which the output of some process within the system is “recycled” and becomes a new input for the system. Feedback can be positive or negative: negative feedback works by reversing the direction of change of some variable; positive feedback increases the rate of change of the variable in a certain direction	Test validation requires a stable market demand	Test validation needs investment. Unless you have a budget that you can plan with – in an uncertain market you’re not going to spend money on test validation. [KI9]
		More lab staff are needed to interpret results but staff who are there and can do the job are less available as they are spending their time training new staff	There’s a huge bottleneck in interpretation of data. There are not enough skilled people. It takes a couple of years to train a scientist. Senior scientists are currently used up in training, supervising and double checking everything. [KI9]
		More staff are needed to make genomic testing in Australia more competitive (than overseas labs) but because they are less competitive, they do not get the business needed to employ more staff	International labs can do the testing at a much lower cost, much faster turnaround time. It makes it hard for hospitals to still go with Australian labs. It’s a feedback loop as they need more business to employ more people to decrease turnaround times. [KI4]

^1^Ellis LA, Churruca K, Braithwaite J. Mental health services conceptualised as complex adaptive systems: what can be learned? *International Journal of Mental Health Systems.* 2017;11:43

^2^Kauffman SA. *The origins of order: Self organization and selection in evolution.* New York: Oxford University Press; 1993

^3^Leykum LK, Lanham HJ, Pugh JA, et al. Manifestations and implications of uncertainty for improving healthcare systems: an analysis of observational and interventional studies grounded in complexity science. *Implementation Science.* 2014;9:165

^4^Plsek PE, Greenhalgh T. The challenge of complexity in health care. *BMJ.* 2001;323(7313):625

^5^De Wolf T, Holvoet T. Emergence versus self-organisation: different concepts but promising when combined. In: Brueckner S, Di Marzo Serugendo G, Karageorgos A, Nagpal R, editors. Engineering self-organising systems ESOA 2004 Lecture Notes in Computer Science, vol 3464. Berlin: Springer; 2005

Interdependencies were described by key informants around barriers to testing. For example, participant KI7 described how consent to testing was dependent on a patient’s understanding and perception of how a test result might affect future insurance premiums or claims. This had negatively impacted recruitment for testing in some Flagship projects, with some people declining as they thought it would compromise their insurance coverage, suggesting it may be a barrier in the future. There were also interdependencies between testing, location and the particular funding model the patient came under that determined access to testing rather than actual clinical need. Unintended consequences were around inaccurate expectations of time (e.g., underestimations of time taken for paperwork, and curation of results), ramifications of limited funding (e.g., leading to inequities of access) and of the technology itself (e.g., making other tests redundant).

Feedback loops represented as circling arrows (e.g., centre of the picture linking clinical laboratories, Functional Genomics and MDT models of care) show how advances in one area can influence others in a positive growth of understandings. These feedback loops were apparent across most of the research clusters. For instance, Ethics within Interdisciplinary Research was formally addressing issues of uncertainty of results, incidental findings and how to manage the informed consent process. This in turn was informing clinical practice, data storage and privacy, education, position statements from peak bodies, and development of things such as a national consent form. Other research such as Health Economics was being used to build the evidence base of clinical utility and effectiveness, which in turn influenced funding and policy decisions.

Features of a CAS were considered for their potential as leverage points or modifiable factors. These are discussed below.

## Discussion

A rich picture of the translational work of Australian Genomics within the wider genomic landscape, was produced through co-design of researchers with health services expertise, key informants with experiential knowledge of working within the system, a systematic review and review of relevant documents and websites. Informants described their experiences and observations of clinical genomics work of Australian Genomics and the wider context in which it operates. From this, features of complex adaptative systems were readily identified by the researchers. The rich picture, as a two-dimensional graphic could not depict all the nuances of complexity which we revealed so is accompanied by the narrative in Table 2.

Clinical genomics is shown to be a highly complex intervention involving many actors and multiple nested systems. The systems perspective applied in this study presents a holistic and nuanced view of processes, influences and interactions. Many studies have examined processes and interactions at the unit level [32] or focused on a specific group of patients [33] or health professionals [34]. Others have looked at global endeavours in genomic medicine [16], describing and contrasting genomic programs in different countries. Useful as these papers are, this to our knowledge, is the first holistic study in which an entire national genomic program and its links and relationships with the broader context is considered. A holistic picture highlights key features that can easily be missed when using a reductionist approach. For example, there is the case of funding for tests wherein all funders intended to increase equity of access to genomic testing for patients. The unintended consequences of the resulting patchwork of state, federal, research and philanthropic funding, coupled with the burden placed on clinicians to seek out one or more of these sources for their patients to be tested, sets up an inequitable system of testing allocation, the overall pattern of which was largely invisible nationwide. This holistic perspective is sorely needed because the evidence for clinical utility of genomic interventions within different conditions is rapidly emerging, and its integration within routine care requires traditionally siloed parts of the healthcare system to work together in new ways.

The development of a rich picture can surface divergent viewpoints influencing the situation [35] that are essential to recognise when attempting to understand the situation. Furthermore, by visualising a complex situation in a picture, it can be viewed and discussed by multiple stakeholders that may have more or less knowledge and understanding about different parts of the system as well as divergent views about the situation. Therefore, rich pictures have the potential to aid discussions that can help stakeholders to conjoin their points of view and increase their understanding of other stakeholders’ perceptions and actions, both which are essential for taking actions to improve a situation [22].

A frequent comment from the Key Informants of this study as they looked at the rich picture was that while they could comment on “their section of the picture” they were not aware of what was going on elsewhere. Upstream and downstream outcomes that influenced or were influenced by their own local processes were also sometimes unexpected. So, for example clinicians were not always aware of issues facing the data managers or education providers. Laboratory scientists were aware of industry drivers and workforce issues but much less around access issues facing consumers. The interdependencies, feedback loops, uncertainties and unintended consequences found in this study show how tightly coupled processes are and indicate that a whole of system approach must be taken to address issues. It will not be sufficient to consider individual components of the larger system in isolation.

### Implications

Funding was the strongest theme throughout the study. Three main implications of funding were revealed: its influence on which patients have access to genomic testing (and the amount of paperwork and effort required to secure funding for different patients’ tests), on the employment of a suitably skilled genomic workforce that is needed, and on the development of test validation and the biotechnology industry in Australia more generally. It is clear that the web of interdependencies around funding mean that even small adjustments to funding for genomic testing will have ramifications throughout the system. Uncertainty of future funding for genomic testing was seen to be mobilising consumer groups to advocate for Medicare funding and meant that the onerous work done by clinicians in searching for funding sources (outside of research funding) for a needed test will only get worse if Medicare rebates are not approved for tests showing clinical utility. While, as one of our informants noted, Medicare funding was imminent for genomic testing for children with intellectual disabilities, this was the only condition being considered at present. The clinical effectiveness of a number of applications of genomic testing has been established in Australian and overseas studies (e.g., 36). However, we argue that funding schemes considering just clinical effectiveness of genomic interventions within individual conditions, do not take into account the funding needs for the wider workforce and infrastructure required when introducing a new complex intervention.

Unintended consequences included the high pressure on senior genetic specialists to mentor more junior staff and to contribute to the data analysis. This pressure on individuals is not sustainable. Learning from this and observing how health professionals have self-organised and adapted to include new roles such as biostatisticians, should inform the genomic teams of the future. Getting the skill mix of senior and junior staff, genomic specialists and generalists (both within clinical and laboratory settings) will be crucial for the sustainability of the model of care going into the future.

Genomic sequencing is heavily dependent on technological advances both now and in the future; from the sequencers to the reagents, to the data curation, sharing, and storage platforms. A key finding from this study was that in the initial design of Australian Genomics Flagships, the biotechnology industry was not consulted. This lack of engagement with local industry placed a burden on the relationship between research and industry. Genomic technology is constantly evolving and improving; its successful adoption and sustainable implementation as part of routine practice means industry partnerships are crucial.

### Strengths and limitations

A static, two-dimensional rich picture has limits to what it can depict. The position of Education is a case in point (just below the middle to the right). It is physically distant from Professional Education Providers (middle right side) but is obviously closely linked. Government is also split: representing government funding on the top left and health funding at the bottom right. Yet another example is the placement of Patients and Public physically distant from the Multidisciplinary teams caring for them (just above the centre to the right). Drawing multiple linkages across all these interacting agents would make the picture too “busy” to understand visually. The same is true of feedback loops which were noted between multiple parts of the graphic.

The graphic depicts the landscape in Australia and so is dominated by its publicly funded health care system (split between Federal and State funding models). However, the list of stakeholders, issues, and the interplay between them will doubtless be informative to other countries. Methods are described in detail so that other research groups can develop their own rich picture. Other strengths are that data was acquired from multiple sources, and the validation process involving nine experts actively involved in different aspects of clinical genomics.

## Conclusion

The rich picture of the clinical genomic landscape in Australia is the first to show as comprehensively as possible the full health system within which genomics is embedded, with key stakeholders, agencies, processes and their interdependencies. A complex systems approach, in which the system is viewed as a dynamic whole, represents a genuine, deep-seated attempt to understand key drivers and barriers to largescale interventions such as clinical genomics. Stakeholders frequently commented that they were familiar with “their part of the system” but not others. In depth studies of individual clinical microsystems, single stakeholder perceptions, and effects of specific policy and funding decisions are important for facilitating understanding but they do not factor in the features inherent in the features inherent in CASs (e.g., interdependencies, non-linear processes, fuzzy boundaries). An overarching understanding of health care as a CAS is important for feasible and pragmatic implementation decisions. In particular, this complexity view revealed multiple sources of funding forming a patchwork of funding models across states. While each source individually was intended to increase access of patients to testing, this had the unintended consequence of adding a burden on clinicians to find funding for individual patients, and contributed to a clearly emerging inequity of access. This complexity informed perspective should be taken into account in future policy decisions.

## Supplementary Information

**Additional file 1.** Interview schedule for eliciting feedback on the rich picture.

## Data Availability

Interview transcripts and selected Australian Genomic documents are not available as per Ethics requirement that we maintain confidentiality of sites and individuals. Interview schedule is provided in the Additional file 1.

## Abbreviations

CAS: Complex adaptive system
KI: Key Informant
