# Supplementary material for: A dynamic systems view of clinical genomics: a rich picture of the landscape in Australia using a complexity science lens
Source: BMC Med Genomics. 2021 Feb 27;14:63. doi: 10.1186/s12920-021-00910-5 (PMC7912922; doi:10.1186/s12920-021-00910-5)
Supplement: Supplementary file 1 — Additional file 1. Interview schedule for eliciting feedback on the rich picture. [file 12920_2021_910_MOESM1_ESM.docx]

Interview schedule for Key Informants of genomic implementation in Australia

**Aim**: To refine and validate rich pictures of national level and health service level (laboratories)

**Materials:**

- Participant information sheet and consent form
- Rich picture draft
- Pencil

Get participant to read and sign consent form. Check they are happy for it to be recorded and remind them their comments will be treated confidentially.

**Introduction**

*Thank you for agreeing to be one of our expert advisors. We are recording this so we can listen to what you are saying and not be distracted by taking notes. It is our intention that you remain anonymous in any reporting.*

Show Rich picture and give participant a pencil. Encourage them to draw on the graphic to add, subtract or move items and to “think out loud” while doing it.

*This is a graphic of features, influences and stakeholders involved in Australian Genomics’ endeavour to introduce genomic medicine into routine care.*

1. *Do you think this is an accurately representation of the national level?*
2. *Have we missed out any components or stakeholders?*
3. *Have we missed out any interactions or influences?*
4. *Are you now happy with it? Would you be happy for us to send you the final draft of the graphic once we get all our experts to refine it, as a final validation?*

*Thank you. We also have three themes that have emerged from the literature and from talking to people that we would like to get your thoughts about.*

1. *The first theme is funding. Can you tell us about funding for genomics from your perspective? [Prompt: Is funding adequate? Does it raise issues of equity of access to testing?]*
2. *The second theme is “new ways of working” in genomics, for example, working with different professionals. Do you have any comments about that?*

*[Prompt: How is genomic work different from other clinical or lab practice? Have any new ways of working impacted your practice, whether it be a positively or negatively?]*

1. *The third theme is around uncertainty, unpredictable outcomes or unintended consequences that arise as one practices genomic medicine. What are your thoughts about that?*

*[Prompt: People talk a lot about the uncertainty of some results and the difficulty of dealing in that uncertain zone, so there’s that aspect. We also wonder if you have ever observed times when unexpected, unpredictable things had happened, or something that seemed perfectly logical was shown to be wrong.]*

*Thank you for your time.*
